# Supplementary material for: Feeding Malic Acid to Chickens at Slaughter Age Improves Microbial Safety with Regard to Campylobacter
Source: Animals (Basel). 2021 Jul 5;11(7):1999. doi: 10.3390/ani11071999 (PMC8300160; doi:10.3390/ani11071999)
Supplement: Supplementary file 1 [file animals-11-01999-s001.zip › animals-1283947-supplementary.pdf]

Supplementary Materials

Table S1. Composition of the feed supplied to the chicken flocks

Items	Content	
	Starter (1-3 week)	Grower (3 week - slaughter)
Ingredient (%)		
Corn	59.74	60.06
Wheat	—	5.40
Soybean meal	25.50	18.50
Cottonseed meal	2.00	3.50
Meat and bone meal	—	2.00
Fish meal	1.50	—
DDGS[1]	—	5.00
Limestone	1.40	0.90
NaCl	0.33	0.35
CaHPO4	1.50	0.60
DL-Methionine	0.20	—
L-Lysine	—	0.40
HMA[2]	0.20	0.13
Threonine	0.03	0.05
Middling	5.00	—
Fat and oil	1.00	1.60
Choline chloride	0.10	0.10
Premix[3]	1.50	1.50
Total	100.00	100.00

1. Distillers dried grains with soluble.

2. Methionine hydroxyl analogue.

3. The premix provided the following per kg of diets: VA 9 200 IU, VD 3 000 IU, VE 38mg, VK_3 3 mg, VB_1 3 mg, VB_2 10 mg, VB_6 5 mg, VB_{12} 0.04 mg, niacinaminde 40 mg, calcium pantothenate 16 mg, folic acid 2 mg, biotin 0.3 mg, Fe 66 mg, Cu 15 mg, Mn 95.4 mg, Zn 96.6 mg, I 0.38 mg, Se 0.41 mg.

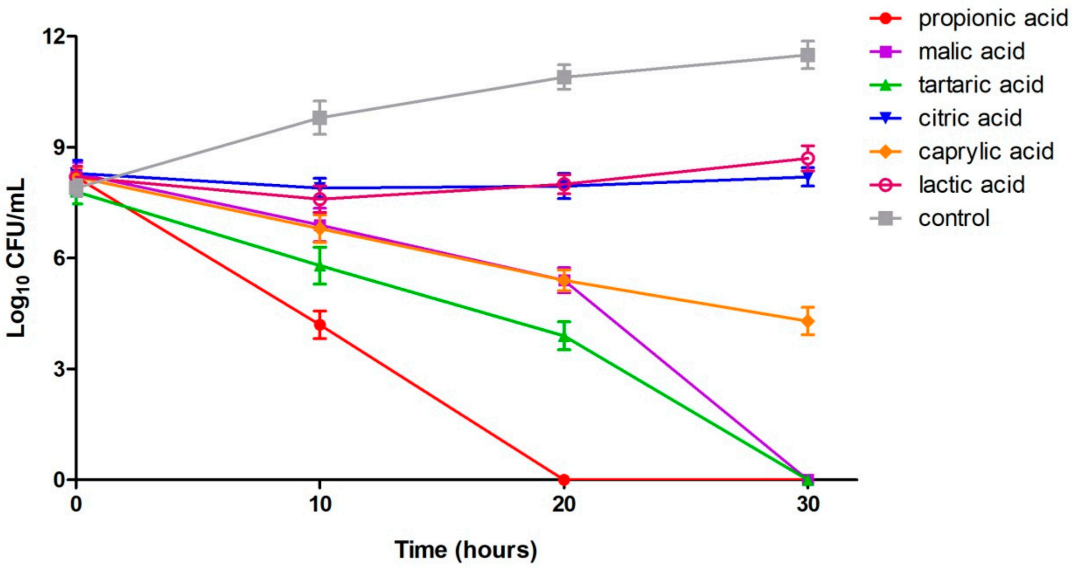

Figure S1. Inhibitory effects of organic acids on the growth of *Campylobacter* in MH broth. The organic acids were added to the MH broth, and the pH was adjusted to 4.0, with approximately 1 x 10^8 CFU/mL *C. jejuni* cultured in this acidified broth for 30 h. Samples were taken at the indicated intervals to determine the bacterial counts. Propionic acid, tartaric acid and malic acid showed good bacteriostatic effects *in vitro* when compared to the other acids.

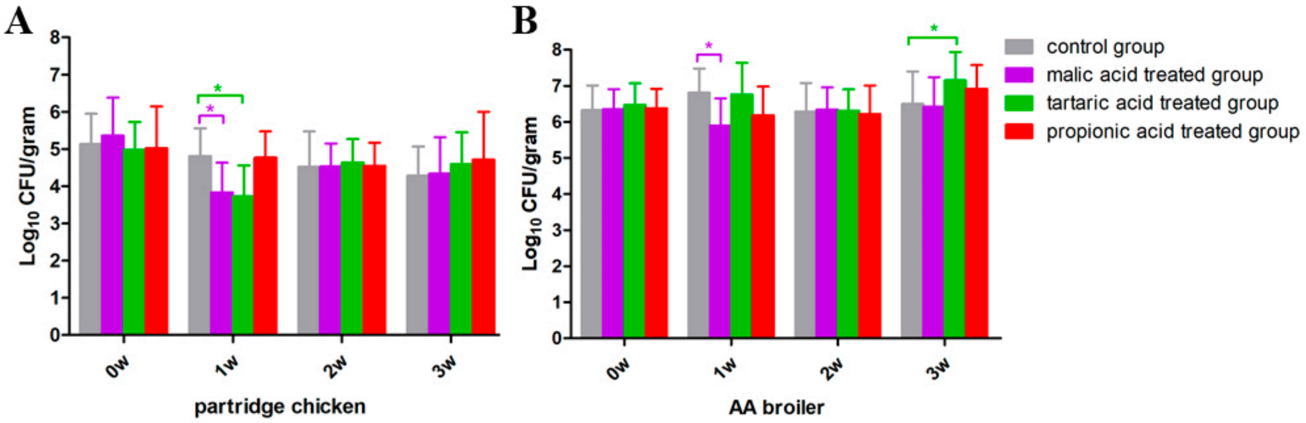

Figure S2. The decontamination effect on *Campylobacter* in chicken flocks by drinking the organic acid-supplemented water. The acids showing good bacteriostatic effects *in vitro* (Figure S1) were supplemented into the drinking water (pH 4.0) of partridge chickens (A) and AA broilers (B). The acidified water was continuously provided for 3 weeks, and the *Campylobacter* carriage was detected by assaying the

cloacal swabs. A stable decontamination effect was found in the malic acid-treated group after one week of use of the acidified drinking water. Twenty chickens were assayed in each group, the data are presented as the mean ± standard deviation (SD), and asterisks with color indicate a significant difference between the corresponding group to the control group ($P < 0.05$ *).
